# Performance of automated CT ASPECTS in comparison to physicians at different levels on evaluating acute ischemic stroke at a single institution in China

**DOI:** 10.1186/s41016-021-00257-x

**Published:** 2021-10-01

**Authors:** Xiaochuan Huo, Raynald, Hailan Jin, Yin Yin, Guangming Yang, Zhongrong Miao

**Affiliations:** 1 Department of Interventional Neuroradiology, Beijing Tiantan Hospital, Capital Medical University, Beijing, 100070 China; 2 Department of R&D, UnionStrong (Beijing) Technology Co. Ltd, Beijing, China

**Keywords:** ASPECTS, Computed tomography, Ischemic stroke, Physicians, Automatic device

## Abstract

**Background:**

Our aim was to evaluate the sensitivity and specificity of the automated computer-based Alberta Stroke Program Early CT Score (e-ASPECTS) for acute stroke patients and compare the result with physicians at different levels.

**Methods:**

In our center, e-ASPECTS and 9 physicians at different levels retrospectively and blindly assessed baseline computed tomography (CT) images of 55 patients. Sensitivity, specificity, receiver-operating characteristic curves, Bland–Altman plots with mean score error, and Matthews correlation coefficients were calculated. Comparisons were made between the scores by physicians and e-ASPECTS with diffusion-weighted imaging (DWI) being the ground truth. Two methods for clustered data were used to estimate sensitivity and specificity in the region-based analysis.

**Results:**

In total, 1100 (55 patients × 20 regions per patient) ASPECTS regions were scored. In the region-based analysis, sensitivity of e-ASPECTS was better than junior doctors and residents (0.576 vs 0.165 and 0.111, *p < 0.05*) but inferior to senior doctors (0.576 vs 0.617). Specificity was lower than junior doctors and residents (0.883 vs 0.971 and 0.914) but higher than senior doctors (0.883 vs 0.809, *p < 0.05*). E-ASPECTS had the best Matthews correlation coefficient of 0.529, compared to senior doctors, junior doctors, and residents (0.463, 0.251, and 0.087, respectively).

**Conclusions:**

e-ASPECTS showed a similar performance to that of senior physicians in the assessment of brain CT of acute ischemic stroke patients with the Alberta Stroke Program Early CT score method.

## Background

Computed tomography (CT) is still the most widely used tool for AIS because it is fast, efficient, easy to access, and reliable to rule out hemorrhage, while others are more time consuming and contraindicated for some patients [1–3]. Despite its advantage compared to other imaging modalities, subsequent research showed that there was still inconsistency between observer to recognize and quantify these changes. Therefore, a useful scoring system, the Alberta stroke program early CT score (ASPECTS), was designed to semi-quantify and describe the topography of cerebral tissue damage caused by AIS [4, 5]. Unfortunately, the interrater variability and modest interobserver agreement which depend on their experiences have become a serious limitation for the scoring methods [6–10]. Hence, an automated software application based on ASPECTS scoring system (e-ASPECTS) was made to enhance the usefulness of CT imaging and to optimize the evaluation of AIS patients. In this study, we compared the scoring performance of e-ASPECTS to those of nine independent physicians with different working experiences.

## Methods

**Fig. 1:**
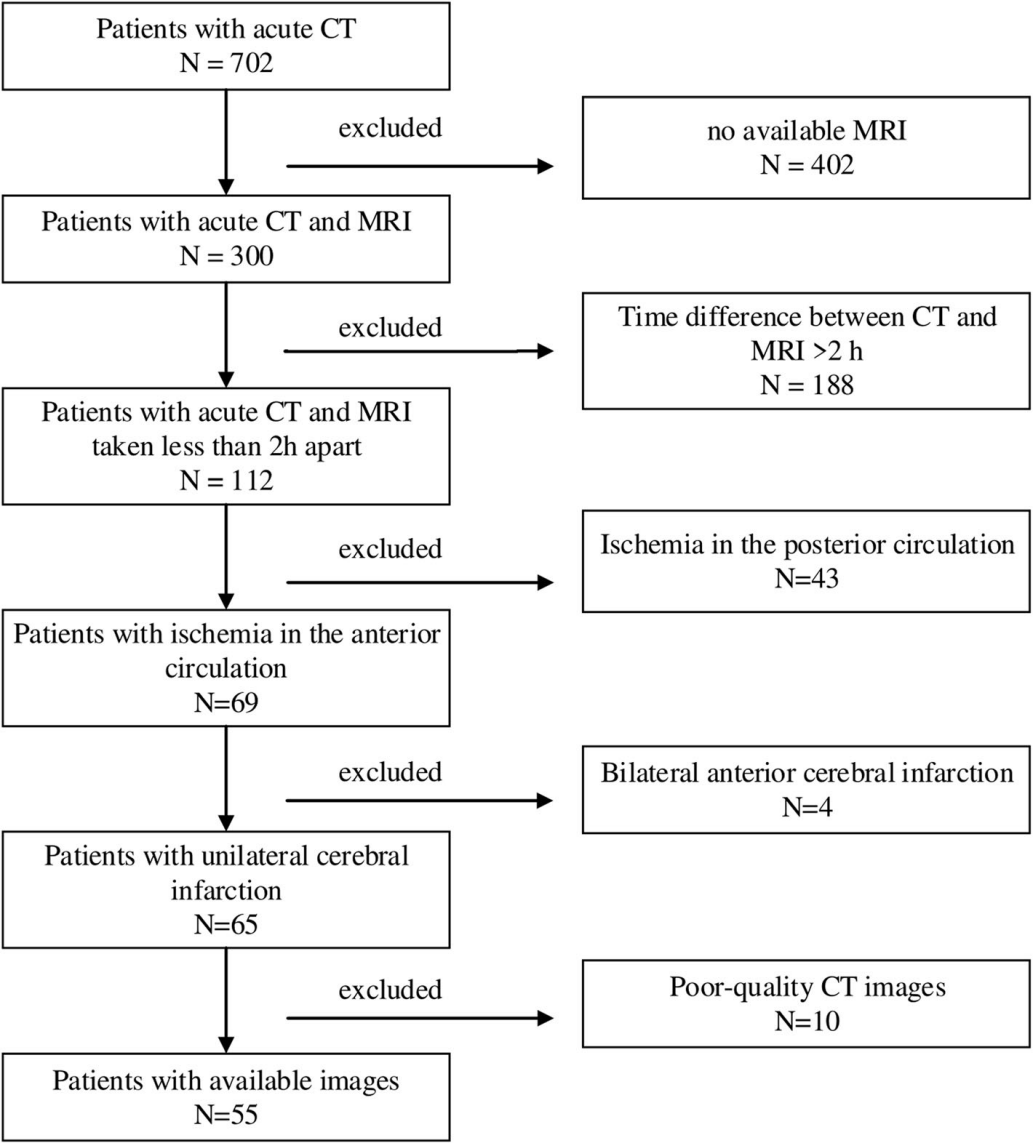
The flow chart of inclusion and exclusion criteria of the patients in the presented study

Fifty-five patients participated in this study (Fig. 1); a CT and an MRI were obtained for stroke diagnosis. The automatic device e-ASPECTS (UGuard V1.0.0.3d9fb70114, Union Strong (Beijing) Technology Co. Ltd, China) and nine doctors interpreted the CT scanners using ASPECTS independently. All physicians were instructed to evaluate the CT in the correct use of the ASPECTS scoring system according to www.aspectsinstroke.com. Scorers were allowed to view the whole brain scan and to scroll the images backward and forward and to adjust the contrast, brightness, window/level, and magnification of the images. The physicians were grouped according to their working experiences. The senior doctors had extensive experiences in neurovascular disease for at least 10 years. The junior doctors’ experiences were varied, with at least 5 years in neurovascular disease. The resident doctors had experiences in neurovascular disease for at least 3 years.

**Fig. 2:**
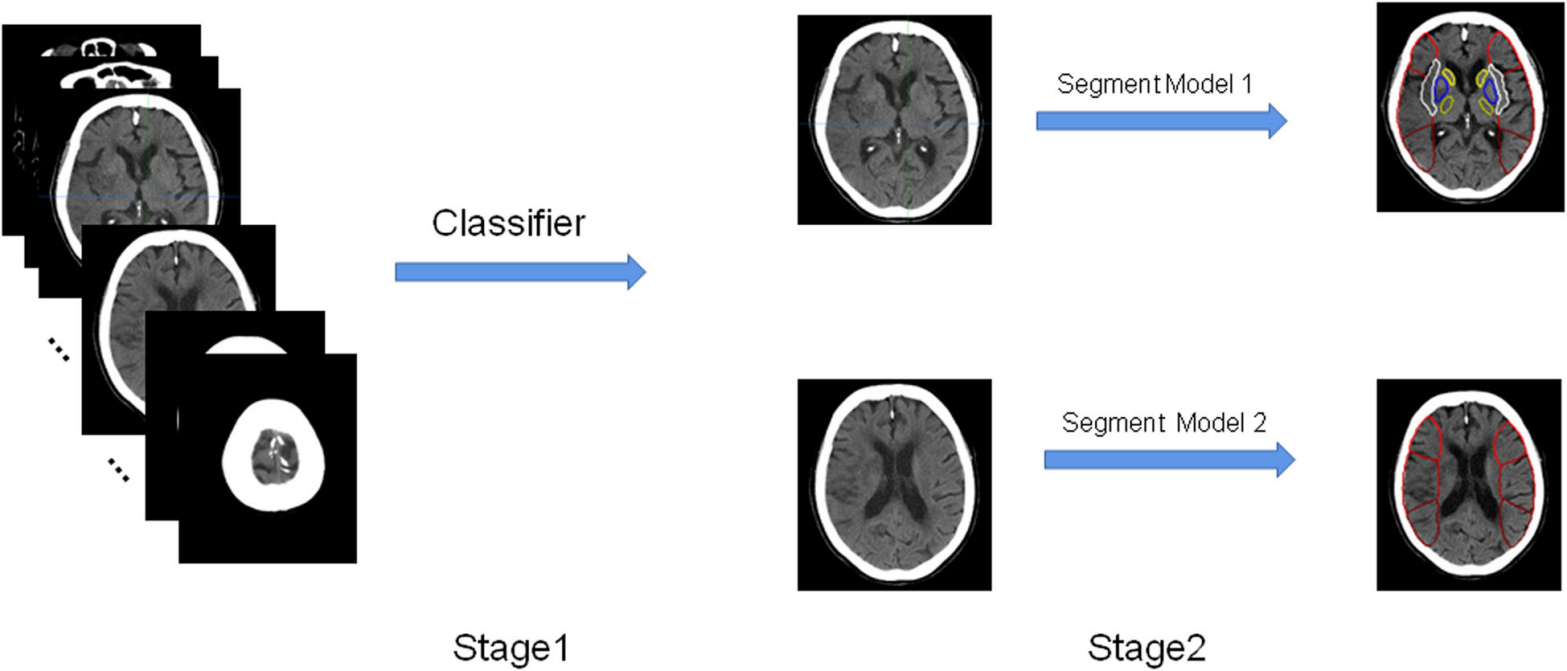
Flow chart of the software analysis

We developed 1 classifier and 2 segmentation models to accomplish the task. The classifier used visual geometry group (VGG) model pre-trained on a large dataset as feature extractor, through which we can fine tune to extract target slices from the CT head series as in stage 1. In stage 2, segment model 1 detects 14 areas in the nucleus mass layer, and segment model 2 detects 6 areas in the nucleus mass upper layer. The segmentation models had the encoder-decoder architecture as U-Net and made better use of features through dense connections (Fig. 2).

The ASPECTS score of the ground truth was determined by Tiantan Hospital Stroke Centre based on medical professional interpretation of the MRI scanner as the gold standard. The ground truth was defined as the ASPECTS on DWI and was scored on consensus basis by two experts who were not blinded to the clinical information. Any detectable DWI lesion attributable to acute cerebral ischemia, independently of its size, was scored within the different ASPECTS regions by assessment of the whole DWI scan. We evaluated the performance of the automatic device by comparing how well the ASPECTS aligned with the ground truth. The study was approved by the committee of ethics in Beijing Tiantan Hospital. All patients were treated according to the stroke management protocols.

The automatic device produces binary scores of 20 regions between the cerebral hemispheres of every patient. The physicians determined the stroke by region as well. The ASPECTS score was calculated by summing up the binary scores of all the regions. We evaluated the alignment between each group and the ground truth using the concordance correlation coefficient (CCC). CCC ranges from − 1 to 1, and a higher value indicates better alignment. We also produced Bland-Altman plots to compare the alignment between each group and the ground truth. The dots in a Bland-Altman plot with a random pattern indicate good alignment between the two methods. Besides, the histograms of the difference in ASPECTS scores were produced to assess measurement accuracy.

As we were interested in diagnosing large infarct core (ASPECTS < 6), we dichotomized ASPECTS using the cut-off ASPECTS < 6 and evaluated the performance of the automatic device as well as the physicians. We illustrated the performance using the receiver-operating characteristic (ROC) curves, where the curve plots showed sensitivity changed relative to specificity. We compared sensitivity as well as specificity between the device and any of the three groups of physicians using a generalized linear mixed-effects model. Specifically, we utilized a logistic regression model. We used a non-inferiority test to examine whether the automatic device was no worse than the physicians by groups. The device was considered as not worse than its counterpart if the sensitivity was at most 0.1 less. Similar criteria applied to the specificity.

Since the lack of efficiency in time was a concern when diagnosing acute stroke, we also estimated the average processing time and compared the result between the device and doctor groups. An analysis of variance (ANOVA) model was used for the comparison. The analysis was performed in R version 3.3.1.

## Results

**Fig. 3:**
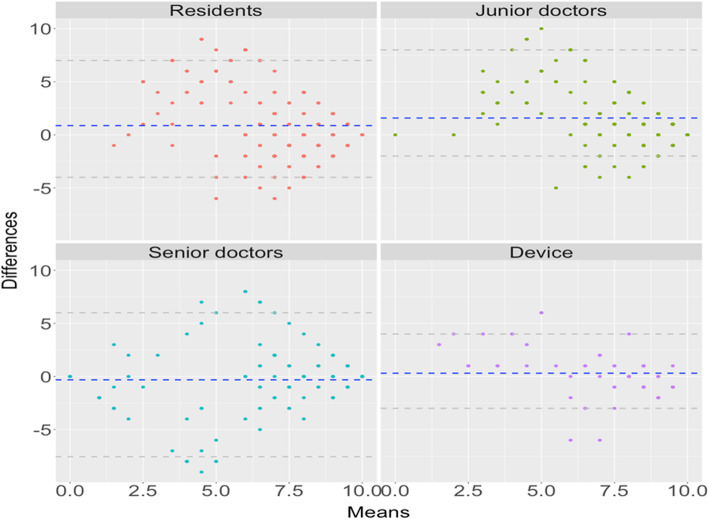
Bland-Altman plots with mean score error (blue dashed line) of score error for the device and doctors in each group

**Fig. 4:**
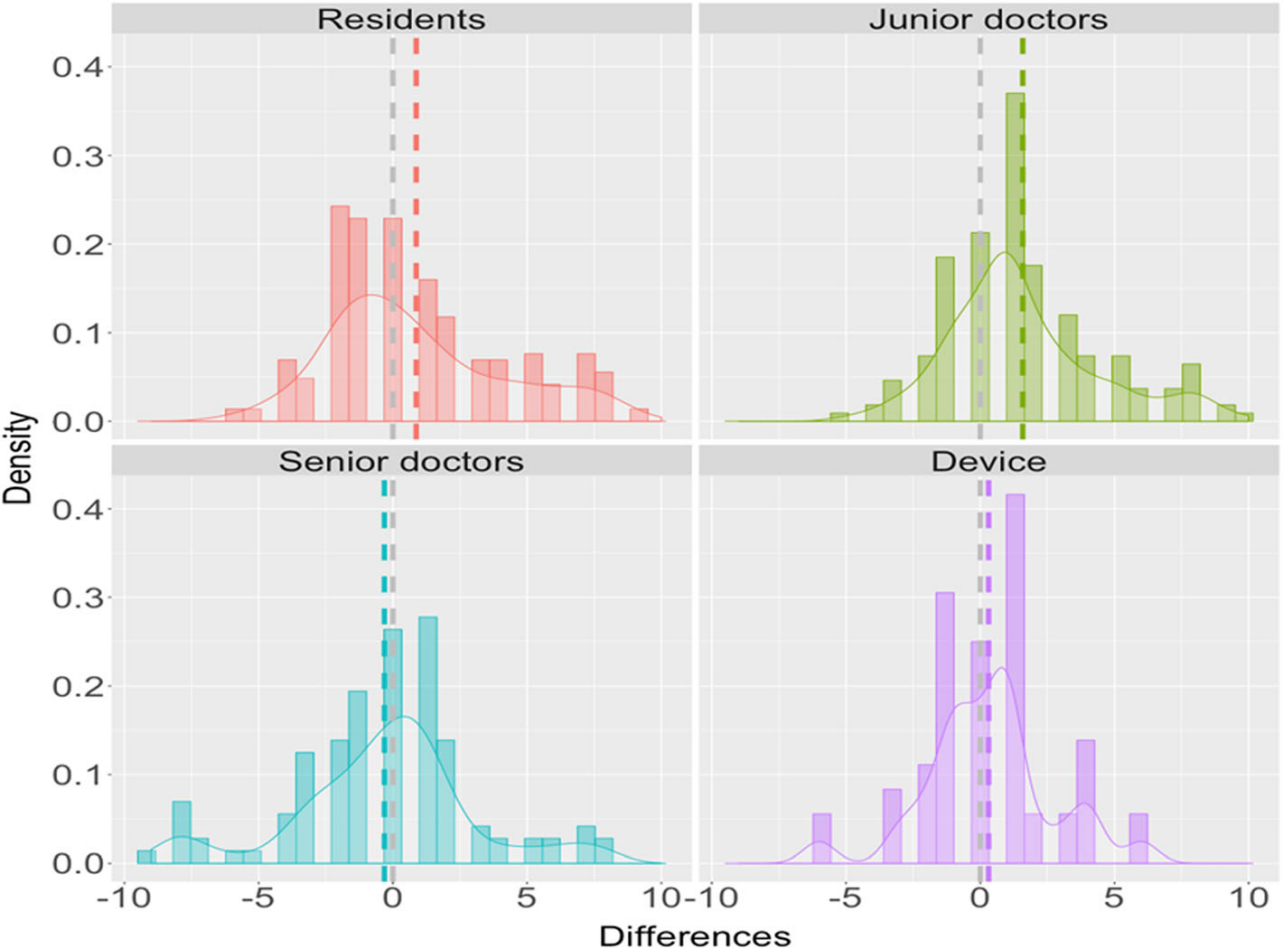
Difference in ASPECTS by group

We summarized the concordance correlation coefficients (CCC) by measurement methods in Table 1. The estimated CCCs, as well as the corresponding 95% confidence interval, were produced. We found the automatic device provided the most accurate ASPECTS score (0.529), followed by the senior doctors (0.463). The ASPECTS score was least accurate in residents group (0.087). Figure 3 summarized the Bland-Altman (BA) plots by group. We found the BA plots for all physician groups showed some systemic patterns while the plot for the device was random. Figure 4 summarized the difference in ASPECTS score between each group and the gold standard. The difference was closer to 0 between the automatic device and the ground truth. These findings indicated that the device was more accurate compared to the physicians in determining the ASPECTS score.

**Table 1:** Summary of concordance correlation coefficient (CCC) and corresponding 95% confidence interval (CI) by group

Rater group	CCC	95% CI
Residents	0.087	(− 0.026, 0.198)
Junior doctors	0.251	(0.142, 0.353)
Senior doctors	0.463	(0.308, 0.594)
Device	0.529	(0.339, 0.678)

**Fig. 5:**
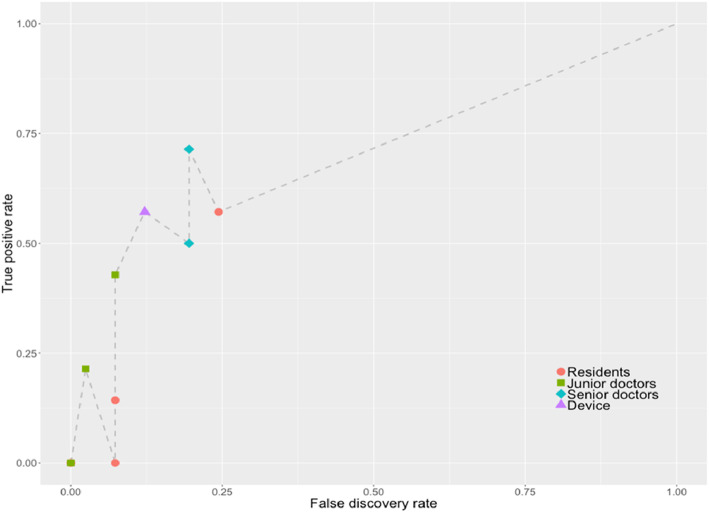
Receiver-operating characteristic (ROC) curves for e-ASPECTS shows performance of the device and every doctor included in this study

The ROC curve was shown in Fig. 5. The automatic device performed better than the residents and junior doctors in correctly detecting the large infarct core; it performed equally well to the senior doctors (0.576 vs 0.617), in terms of sensitivity. We also found the specificity to be higher with the device, compared to the senior doctors (0.883 vs 0.809), while being slightly lower than the residents (0.883 vs 0.914) and junior doctors (0.883 vs 0.971). Besides, sensitivity in the automatic device was no worse than that with the residents (0.576 vs 0.111) as well as the junior doctors (0.576 vs 0.165) at 0.05 significance level; specificity in the automatic device was at least as good as that with the senior doctors at 0.05 significance level (Table 2).

**Table 2:** Sensitivity and specificity by group

Rater group	Sensitivity	Specificity
Residents	0.111*	0.914
Junior doctors	0.165*	0.971
Senior doctors	0.617	0.809*
Device	0.576	0.883

*P* value is pairwise non-inferiority test with a difference of 0.1 between the device and each group of physicians; *the device is non-inferior than the corresponding group of doctors in diagnosing the large infarct core at significance level of 0.05

**Fig. 6:**
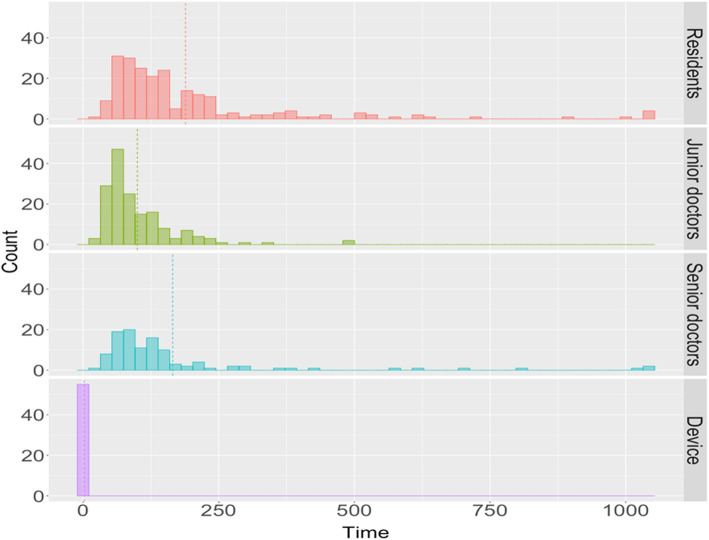
Histogram of processing time by group; the dashed line is the mean processing time by group

**Fig. 7:**
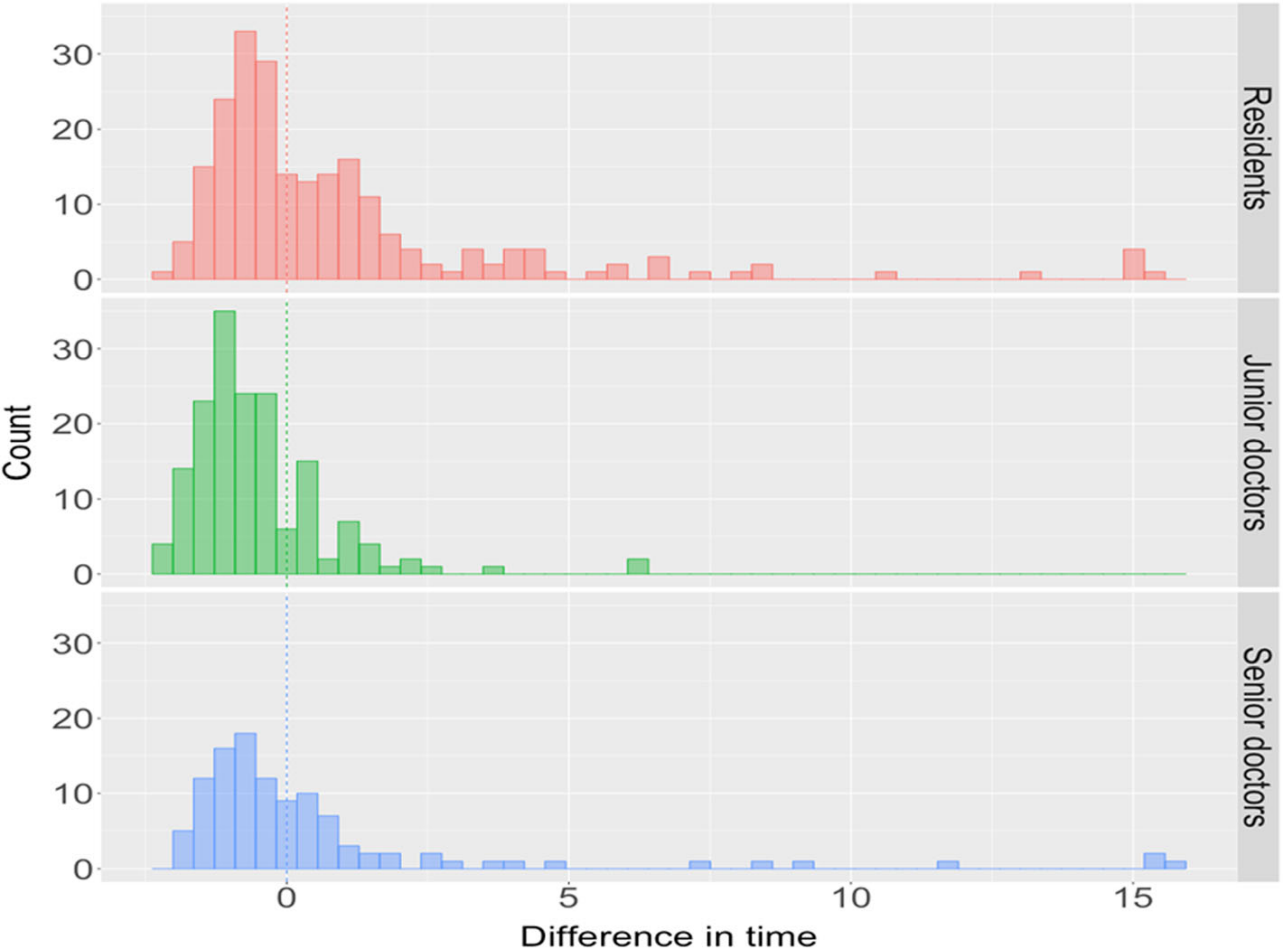
Histogram of difference of processing time, between doctors and the device, by group

The mean and standard deviation of processing time were summarized in Table 3. We also illustrated the processing time by group in Fig. 6. In addition, the difference in time between the group of doctors and the device was summarized in Fig. 7. We found that the junior doctors needed the least processing time on average, while the residents took longer time (3.148 min, mean average). However, the processing time was not statistically significant among other groups (2.193 s, 2.753 min, and 1.667 min, for device, senior doctors, and junior doctors, respectively).

**Table 3:** Summary of processing time by group

Rater group	Time (minutes)
	Mean (SD)
Residents	3.148 (3.085)
Junior doctors	1.667 (1.162)
Senior doctors	2.753 (3.261)
Device	2.193 (0.317)

## Discussion

In the developing country, different cities provide different quality of medical service. Since the devices are not well-distributed, they can affect the treatment outcome [11]. In acute ischemic stroke (AIS), it is important to achieve optimal and good functional outcome in early reperfusion [12–15]. This has to be supported by the assessment device that provides less time and revealed AIS accurately. CT perfusion (CTP), magnetic resonance imaging (MRI) perfusion, and diffusion weighted imaging (DWI)/fluid attenuation inversion recovery (FLAIR) mismatch have been suggested prior to reperfusion [16–21]. However, CT is still the most widely used tool for AIS because it is fast, efficient, easy to access, and reliable to rule out hemorrhage, whereas others are more time-consuming and contraindicated for some patients [22–24]. The capability of doctors in hospital plays an important role in supporting the treatment. Although CT can reveal a significant change of AIS signs such as edema and hypoperfusion [25], not all doctors in developing and developed country are sensitive and aware of these clinical changes [2, 26], particularly those who are not specialized in stroke care diagnosis and management. Therefore, a machine-learning method aiming to help doctors make better clinical decisions is urgently needed and should be disseminated worldwide. Consequently, patients with AIS will benefit from the corresponding treatment.

Our current study aimed to evaluate the performance of an automated software application based on ASPECTS scoring system (e-ASPECTS) in assessing patients with AIS. We compared e-ASPECTS to physicians with different experiences from the same center. Our study showed that e-ASPECT performance was not only as good as the less-experienced doctors, but also not inferior to the senior doctors. The findings were further supported by the Bland-Altman plots and CCC. Both the relatively random pattern in the BA plots and the higher values in CCC (0.529) suggested good performance of the device. From this result, e-ASPECTS provided the best agreement with the ground truth data as compared to physicians. Specificity of e-ASPECTS was lower than senior doctors, but higher than junior doctors and residents, because these two groups were less sensitive. The difference in specificities was significant although it was quite small; it might be due to the unaffected regions that the majority of the data predominated when calculating the specificity. Meanwhile, the differences in sensitivities were quite large. The sensitivity of e-ASPECTS was lower than senior doctors, but significantly higher than residents and junior doctors. This result suggested that physicians with different experiences perform differently, i.e., a relatively large increase in sensitivity at a cost of small decrease in specificity for senior doctors. Besides, the device required least processing time in e-ASPECTS when analyzing ischemic area, compared to other groups. Although e-ASPECTS was superior in processing time when analyzing ischemic changes, this result was only considered as a reference. However, this result showed that in the future e-ASPECTS could be suitable in supporting early diagnosis and management of acute ischemic case, in order to achieve good prognosis.

Our finding with respect to sensitivity and specificity is in accordance with previous reported literatures [1, 2, 26–28]. This electronic ASPECTS could be suitable to be applied in routine diagnosis of acute ischemic stroke because it is CT based, which is widely used in most institutions. Even in recent study, it has been used to predict the prognosis of acute ischemic stroke patients undergoing endovascular reperfusion therapies. Research shows that it can provide important technical support in estimating patient’s prognosis [29, 30]. Moreover, CT is not only capable to shorten time management due to its easy accessibility and fewer physical limitations compared to MRI, but it is also more widely available in most hospitals [24]. On the basis of these studies, the application of e-ASPECTS should be promoted more in clinical routine, especially in developing countries, where medical services and quality are not well-distributed, especially in the rural area. It requires at least 5 years of training to get familiar with diagnosing neurovascular diseases for physicians, while the incidence of acute ischemic stroke is increasing. Thus, more applications of electronic-based ASPECTS are urgently needed. It should be noted that the aim of e-ASPECTS is not to replace expert assessment of the scan or junior physicians, but instead to assist them in clinical routine and research. Although the objectivity of e-ASPECTS was higher compared to physicians, the pre-existing conditions and various changes in human brain are still challenging for the computer-aided ASPECTS assessment because the software is only capable to distinguish acute ischemic changes, and incapable to differentiate the etiologies of brain tissue damage. Hence, a check for plausibility is still needed by the physicians.

In summary, e-ASPECTS showed non-inferiority in applying the ASPECTS to acute ischemic changes compared to experienced doctors and superior compared to moderately and less experienced doctors. Despite this promising result, our study has had several limitations, such as restricted number of patients and lack of generalization as a single center study. A future study involving multiple centers in different regions is in preparation. Besides, the physicians participated in this study were mainly neurologists; more physicians from different departments, such as neuroradiology, neurosurgery, emergency room, and intensive care, should be included. Another limitation was that we did not evaluate the prognosis of patient with or without endovascular treatment based on the e-ASPECTS score. Further studies are required to verify the performance of e-ASPECTS in clinical routine.

## Conclusions

E-ASPECTS performance was shown to be non-inferior to senior physicians and better than junior physicians in assessing the ASPECTS score of AIS patients. E-ASPECTS should be widely applied in routine diagnosis of AIS.

## Data Availability

All data are available to researchers on request for purposes of reproducing the results or replicating the procedure by directly contacting the corresponding author.

## Abbreviations

ASPECTS: Alberta Stroke Program Early CT Score
CT: Computed tomography
CTP: CT perfusion
DWI: Diffusion-weighted imaging
FLAIR: Fluid attenuation inversion recovery
ROC: Receiver-operating characteristic
MRI: Magnetic resonance imaging
VGG: Visual geometry group
